# Reciprocal connectivity between secondary auditory cortical field and amygdala in mice

**DOI:** 10.1038/s41598-019-56092-9

**Published:** 2019-12-23

**Authors:** Hiroaki Tsukano, Xubin Hou, Masao Horie, Hiroki Kitaura, Nana Nishio, Ryuichi Hishida, Kuniyuki Takahashi, Akiyoshi Kakita, Hirohide Takebayashi, Sayaka Sugiyama, Katsuei Shibuki

**Affiliations:** 1 Department of Psychiatry, The University of North Carolina at Chapel Hill, 116 Manning Drive, Chapel Hill, NC 27599 United States; 2 Department of Neurophysiology, Brain Research Institute, Niigata University, 1-757 Asahimachi-dori, Chuo-ku, Niigata, 951-8585 Japan; 3 Laboratory of Neuronal Development, Graduate School of Medical and Dental Sciences, Niigata University, 1-757 Asahimachi-dori, Chuo-ku, Niigata, 951-8510 Japan; 4 Department of Nursing, Niigata College of Nursing, 240 Shinnancho, Joetsu, 943-0147 Japan; 5 Department of Pathology, Brain Research Institute, Niigata University, 1-757 Asahimachi-dori, Chuo-ku, Niigata, 951-8585 Japan; 6 Department of Physiology, The University of Tokyo School of Medicine, 7-3-1 Hongo, Bunkyo-ku, Tokyo, 113-0033 Japan; 7 Division of Otolaryngology, Graduate School of Medicine and Dental Sciences, Niigata University, 1-757 Asahimachi-dori, Chuo-ku, Niigata, 951-8510 Japan; 8 Division of Neurobiology and Anatomy, Graduate School of Medicine and Dental Sciences, Niigata University, 1-757 Asahimachi-dori, Chuo-ku, Niigata, 951-8510 Japan

**Keywords:** Cortex, Neural circuits

## Abstract

Recent studies have examined the feedback pathway from the amygdala to the auditory cortex in conjunction with the feedforward pathway from the auditory cortex to the amygdala. However, these connections have not been fully characterized. Here, to visualize the comprehensive connectivity between the auditory cortex and amygdala, we injected cholera toxin subunit b (CTB), a bidirectional tracer, into multiple subfields in the mouse auditory cortex after identifying the location of these subfields using flavoprotein fluorescence imaging. After injecting CTB into the secondary auditory field (A2), we found densely innervated CTB-positive axon terminals that were mainly located in the lateral amygdala (La), and slight innervations in other divisions such as the basal amygdala. Moreover, we found a large number of retrogradely-stained CTB-positive neurons in La after injecting CTB into A2. When injecting CTB into the primary auditory cortex (A1), a small number of CTB-positive neurons and axons were visualized in the amygdala. Finally, we found a near complete absence of connections between the other auditory cortical fields and the amygdala. These data suggest that reciprocal connections between A2 and La are main conduits for communication between the auditory cortex and amygdala in mice.

## Introduction

The auditory cortex is a center of auditory processing, and it has been found to have a functional map^1,2^. Recent optical imaging studies have indicated that the auditory cortex of the mouse brain is composed of at least four tonotopic subfields^3^ that were tentatively annotated as the dorsomedial field (DM), anterior auditory field (AAF), primary auditory cortex (A1), and secondary auditory field (A2) in a previous report^4^. Subregions in the mouse auditory cortex may be functionally specialized^5–7^, and so connective patterns with other brain regions may vary according to subregion. To date, several neuronal tracing investigations have examined the afferents and efferents to/from the mouse auditory cortex. However, the injection sites have usually been set in A1. Indeed, few investigations have employed systematic injections.

Associations between auditory information and emotional meaning are biologically important because they aid in the detection of upcoming risks. The amygdala is an essential region in establishing associations between tones and emotions^8–11^. Thus, full characterization of the connective pattern between the auditory cortex and amygdala is likely to produce meaningful information. To date, many studies have described the feedforward auditory–amygdala pathway that originates in the auditory cortex^12–15^ and auditory thalamus^12,16–20^. Among amygdala subdivisions, the lateral amygdala (La) is thought to be important for the convergence of auditory cues and footshock information^21^ because La receives afferents from a wide range of sensory systems including the auditory cortex^22,23^. Recent studies characterized a feedback pathway from La to the auditory cortex in mice^24,25^, which were consistent with the finding of older studies that used monkeys^26,27^ and rats^28^. However, neuronal tracing is often conducted without clear segregation between the auditory cortical subregions. As a result, whether the whole auditory cortex or just a local area has reciprocal connections with the amygdala is unclear. This could be addressed via systematic projection mapping between the auditory cortex and the amygdala. In the current study, we visualized comprehensive connectivity between the auditory cortex and the amygdala by injecting cholera toxin subunit b (CTB), a bidirectional tracer, into four tonotopic regions of the auditory cortex, according to a map that was functionally determined using flavoprotein fluorescence imaging^4^. We demonstrated that the A2 is specifically connected with the amygdala, which is consistent with the idea that A2 is a higher-order region in the mouse auditory cortex.

## Results

### Auditory cortical region-specific communications with the amygdala

**Figure 1:**
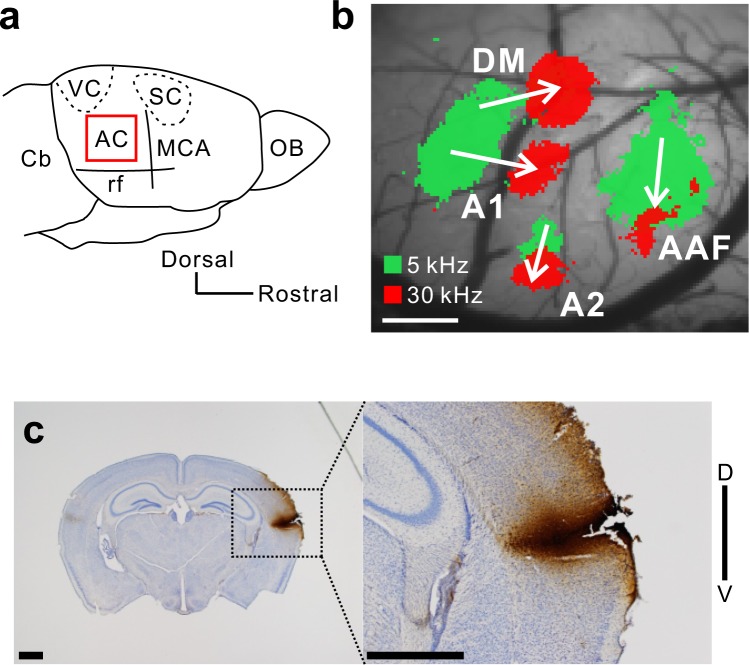
Injection of neural tracers into auditory regions. (**a**) An illustration of the right auditory cortex in mice. AC, auditory cortex; Cb, cerebellum; MCA, medial cerebral artery; OB, olfactory bulb; rf, rhinal fissure; SC, somatosensory cortex; VC, visual cortex. (**b**) Identification of auditory cortical regions with the guidance of tonal responses via flavoprotein fluorescence imaging. Scale bar, 500 μm. AAF, anterior auditory field; A1, primary auditory cortex; A2, secondary auditory field; DM dorsomedial field. (**c**) An example showing CTB injection sites after preparation of coronal slices. Scale bar, 1 mm.

Although there is much debate over how to map the mouse auditory cortex^29^, it is generally accepted that the mouse auditory cortex includes at least four tonotopic regions^3,4^. Here, we refer to them as DM, AAF, A1, and A2 (Fig. 1a,b). After identifying the precise location of AAF, A1, A2, and DM using flavoprotein fluorescence imaging, we injected CTB into the center of the responsive region to examine connective patterns with the amygdala. Deposits of CTB localized within the injected regions were confirmed after preparing brain slice sections (Fig. 1c).

**Figure 2:**
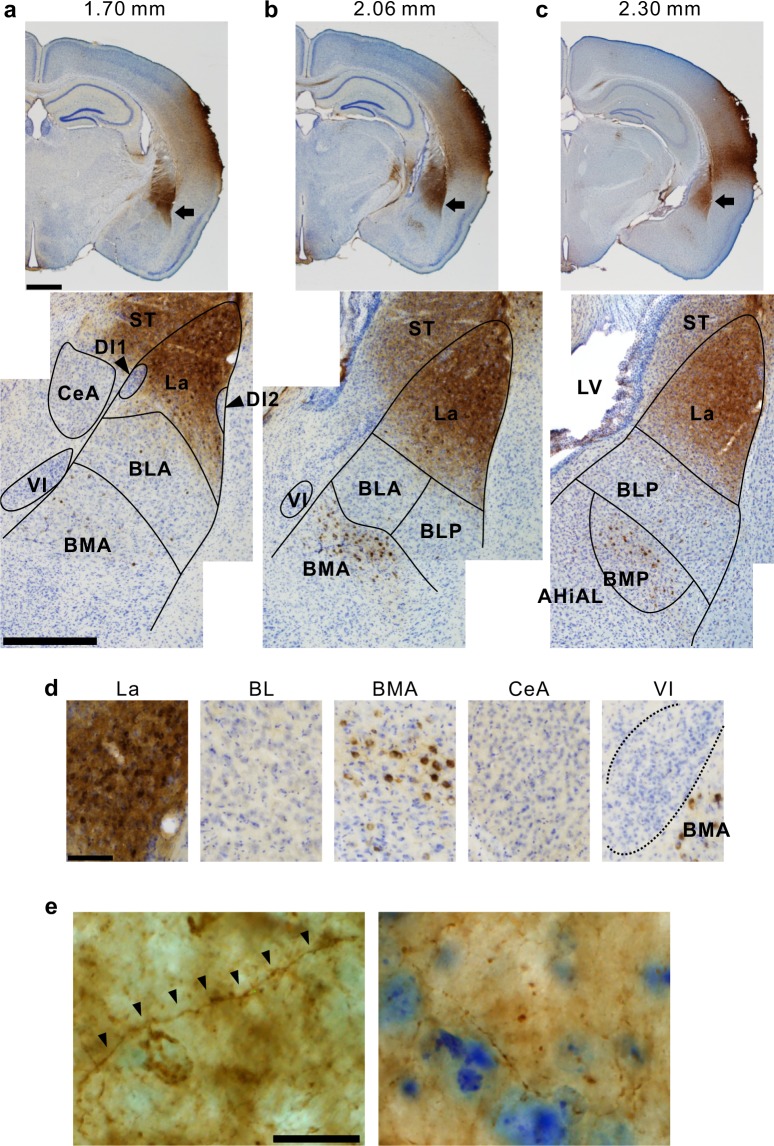
CTB-positive neurons and axon terminals in communication with the auditory cortex. (**a**) Low and middle magnification images of the amygdala 1.70 mm posterior to the bregma. Arrows indicate the CTB-positive amygdala neurons. Scale bar, 1 mm (top) and 500 μm (bottom). (**b**) A pair of images taken 2.06 mm posterior to the bregma. (**c**) A pair of images taken 2.30 mm posterior to the bregma. The arrow head indicates CTB-positive neurons in the higher-order visual thalamus–lateral posterior thalamus (LP). (**d**) High magnification images of subdivisions of the amygdala. Scale bar, 100 μm. (**e**) Example images to show synaptic varicosities in LA which are formed by the axon terminals projecting from A2. Scale bar, 20 μm. AHiAL, anterolateral amygdalohippocampal area; BL, basolateral amygdala; BLA, anterior BL; BLP, posterior BL; BMA, anterior basomedial amygdala; BMP, posterior basomedial amygdala; CeA, central amygdala; DI, dorsal intercalated nucleus; La, lateral amygdala; LV, lateral ventricle; ST, striatum; VI, ventral intercalated nucleus.

**Figure 3:**
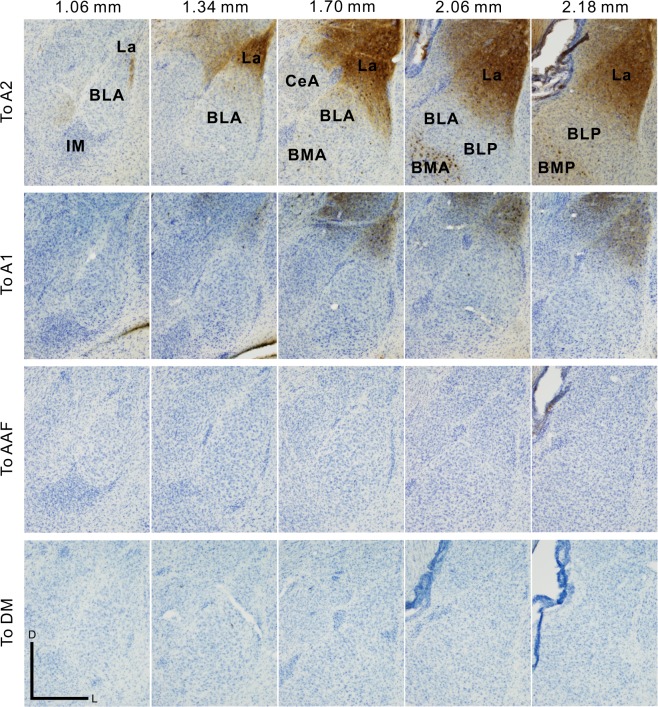
A2- and A1-specific communications with the amygdala. The panel shows the amygdala in the coronal slices after CTB was injected into A2, A1, AAF, or DM in the auditory cortex. IM, main intercalated nucleus. Scale bar, 500 μm.

**Figure 4:**
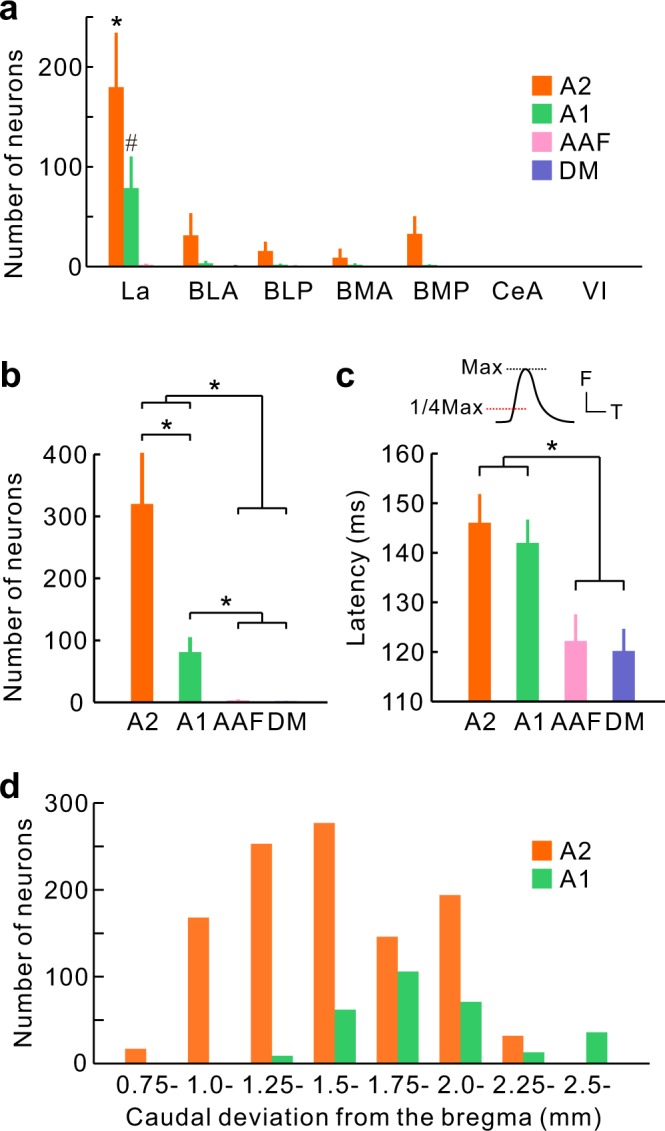
Quantitative analysis of connections between the auditory cortex and the amygdala. (**a**) Quantitative analysis of the distribution of CTB-positive neurons in the amygdala. We found more CTB-positive neurons in La compared with the other subdivisions (*p < 0.001, ^#^p < 0.0001, Dunnett’s test). Data were obtained from five, four, three, and four mice for A2, A1, AAF, and DM, respectively. (**b**) Number of CTB-positive neurons in the amygdala by injection site. *p < 0.05, Wilcoxon signed-rank test after the Bonferroni correction. (**c**) Hierarchical relationships between auditory cortical regions, represented via differences in latencies. Latency represents the time that is required to reach 1/4 maximum of the peak response. The graphs were obtained from the same dataset published in Tsukano *et al*., 2015 (n = 18 mice each; *p < 0.01, Wilcoxon signed-rank test after the Bonferroni correction). F, fluorescence; Max, maximum; T, time. (**d**) Distribution of CTB-positive neurons projecting to A2 and A1 in the rostrocaudal axis. Two distributions were significantly different (p < 10^−6^, chi-squared test). The same sets of brain slices were used for (**a**), (**b**), and (**d**).

We examined the distribution of CTB-positive axon terminals projected from the auditory cortex in the amygdala. The images shown in Fig. 2 were obtained by injecting CTB into A2. We found that densely distributed CTB-positive axon terminals were located mainly in La, and identified slight innervations in other divisions such as the central amygdala (CeA) and basal amygdala (BA), including the anterior basolateral amygdala (BLA), posterior basolateral amygdala (BLP), anterior basomedial amygdala (BMA), and posterior basomedial amygdala (BMP) (Fig. 2a–d). In the high-magnification images, we confirmed that the axon terminals projecting from A2 form many buttons and synaptic varicosities in La, which suggests that the projections from A2 neurons terminate in La (Fig. 2e). As for the three intercalated nuclei, which are a cluster of GABAergic neurons, the two dorsal nuclei the sides of La included some CTB-positive terminals. This is consistent with a previous study^15^. The most well-known cluster, which was located ventrally, included no CTB-positive axon terminals. These data suggest that La mainly receives input from the auditory cortex, and this result is consistent with the well-known view that La is an entrance in the amygdala for sensory input from sensory cortices^11^. Moreover, we found many CTB-positive somata, located mainly in La, after injecting CTB into A2 (Figs. 2 and 3). We quantitatively analyzed the number of CTB-positive somata in each amygdala division (Fig. 4a). Numerous CTB-positive neurons were distributed in La, and we found a small number of neurons in the basolateral and basomedial amygdala. Importantly, we found no CTB-positive somata in CeA (Fig. 4a), despite the proposed role of CeA as the output center of the amygdala^8^. We also conducted anterograde tracing by injecting a tracer into LA and confirmed that a number of axon terminal buttons were visualized in A2 (Supplemental Fig. 1), supporting the presence of projections from LA to A2. These data confirm that A2 projects mainly to La and receives direct feedback projections from La but not from CeA.

We remarked that the density of CTB-positive axon terminals and the number of CTB-positive neurons in the amygdala differed by injection site in the auditory cortex. As described above, A2 has strong connections with La (Figs. 3 and 4a,b). After injecting CTB into A1, we found one third the number of CTB-positive neurons compared with that observed after injecting CTB into A2 (Figs. 3 and 4a,b). The amount of CTB-positive axons was proportional to the number of CTB-positive neurons, indicating that A1 also had few reciprocal connections with La. Furthermore, we did not find CTB-positive axon terminals or CTB-positive neurons inside the amygdala after injecting CTB into AAF and DM (Figs. 3 and 4a,b). Although few CTB-positive neurons or axons were visualized in the amygdala after injecting CTB into AAF and DM, it was not due to a failure in our injection. As a positive control, a number of CTB-positive neurons were visualized in the auditory thalamus of the medial geniculate body (MGB) in the same animals as used in Fig. 3 (Supplemental Fig. 2), indicating a successful injection. Interestingly, the numbers of CTB-positive neurons in the amygdala increased in proportion with the hierarchical order of the injected regions. This was reflected in the tonal response latencies. The quantitative latency data of each subregion were calculated based on our previous study^30^, in which latency was measured as quarter-maximal latencies of sound-induced flavoprotein fluorescence responses (Fig. 4c). Although the latency of flavoprotein fluorescence imaging is longer than electrophysiological methods, the auditory subregions are activated largely in the same order between the two methods^1,30^. These data indicate that A2 is reciprocally connected with La and that it is a main conduit for communication with the amygdala, while A1 has connections that are similar but much weaker in strength.

### Two independent streams between the auditory cortex and La

**Figure 5:**
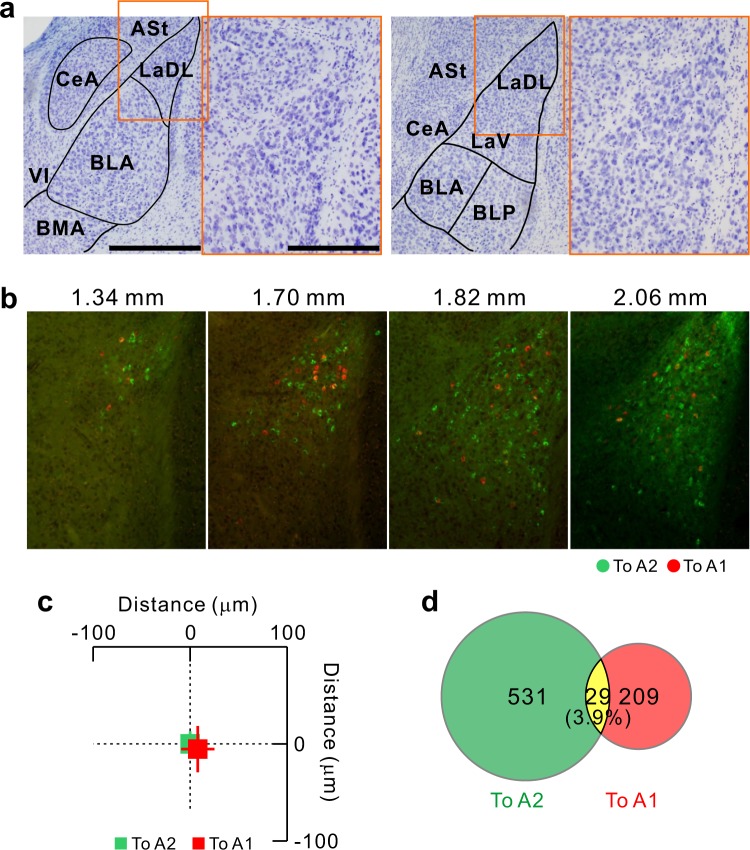
Mutually exclusive La neurons projecting to A1 and A2. (**a**) Coronal views of LaDL 1.58 mm (left) and 1.94 mm (right) posterior to the bregma. The high magnification images show the regions enclosed by the orange rectangles in the low magnification images. Scale bars, 500 μm in the high magnification images and 250 μm in the low magnification images. ASL, amygdalostriatal transition; LaDL, dorsolateral part of the La; LaV, ventral part of La. (**b**) Distribution of La neurons projecting to A2 (green) and A1 (red). Scale bar, 250 μm. (**c**) No spatial topography in neurons projecting to A1 and A2. The averaged coordinates of neurons projecting to A2 were set as the origin. The slices on which both neuronal groups were visualized were used for analysis. We found no significant topographic shifts in the horizontal (p > 0.8; Mann–Whitney U-test) or vertical axes (p > 0.4; Mann–Whitney U-test). Data were obtained from two mice; to A2, n = 282 neurons; to A1, n = 102 neurons. (**d**) Proportion of neurons projecting to the A2 and/or A1. Data were obtained from four mice; to A2, n = 531 neurons; to A1, n = 209 neurons.

Many deep brain parts have initially been considered to be single structures and are then found to actually contain several substructures that can be classified according to relationships with cortical targets^31,32^. To determine whether the origins of the La–A2 and La–A1 pathways are the spatially similar or different inside La, we evaluated the locations of La neurons projecting to A2 and A1. We found that amygdala neurons projecting to A2 were widely distributed rostrocaudally, while those projecting to A1 were comparatively localized in a caudal region (Fig. 4d). However, the distributions of amygdala neurons projecting to A2 and A1 were largely overlapped rostrocaudally. Therefore, we conducted double-injection of two fluorescence CTBs into A2 (green) and A1 (red) to investigate whether La neurons projecting to A1 and A2 were intermingled or topographically-segregated in the coronal view (Fig. 5a). We found that the two neuronal groups were distributed in a salt-and-pepper fashion and exhibited no clear spatial separation (Fig. 5b,c). However, most neurons were labeled with either tracer, and only 3.9% neurons were double-labeled (Fig. 5d). These data indicate that, although La is a single structure containing neurons with different cortical targets that are intermingled, the La–A2 and La–A1 pathways are mutually exclusive and mediated by discrete populations of amygdala neurons.

**Figure 6:**
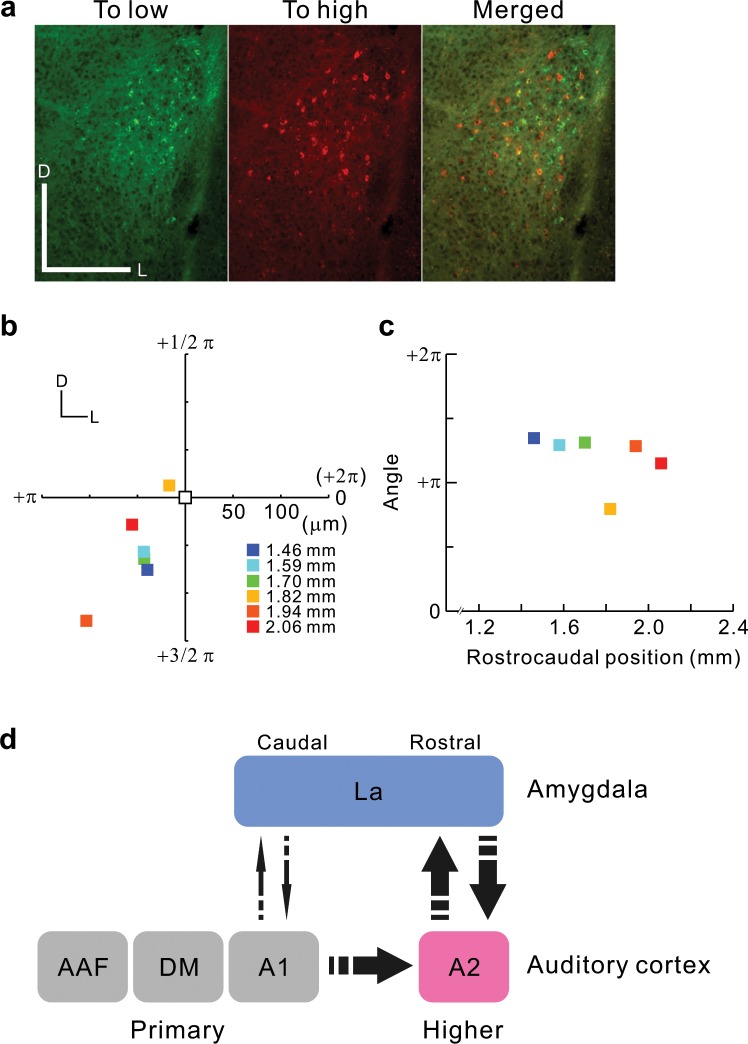
Frequency-related topography in projections between La and A2. (**a**) Example slices showing La neurons projecting to low and high frequency areas of A2. fCTB-positive somata are plotted in the right panels in reference to real images shown on the left. Scale bar, 250 μm. (**b**) Relative locations of La neurons projecting to low and high frequency areas of A2. The averaged coordinates of neurons projecting to low frequency area of A2 are shown in white and set as the origin, and those projecting to high frequency areas are shown in the same Cartesian coordinate system in color. Colors signify rostrocaudal levels of coronal slices in reference to the bregma. Azimuths of color plots are also shown in radians in the polar coordinate system, in which one round corresponds to 2π radians starting from the positive part of the horizontal axis. Data were obtained from four mice. Numbers of neurons were 59 at 1.46 mm, 48 at 1.58 mm, 187 at 1.70 mm, 93 at 1.82 mm, 15 at 1.94 mm, and 67 at 2.06 mm posterior to the bregma. (**c**) Correlations between rostrocaudal location and azimuths in (**b**), r = 0.77, p > 0.07, Spearman’s test. (**d**) Summary of the current results showing the connectivity between the auditory cortex and amygdala in the mouse brain.

The auditory cortex has tonotopic organization based on tonal frequencies. It is already known that the auditory cortex is connected to several deep brain structures in a frequency-related, topographical fashion, as indicated by auditory cortico-striatum projections^33^. To examine whether La and A2 are connected topographically, we injected two fluorescent CTBs into low and high frequency areas of A2 (Fig. 6). Although the distribution of the two neuronal groups was wider and more intermingled, the averaged locations of the two neuron types were significantly shifted in the dorsoventral direction on all rostrocaudal levels (Fig. 6b,c). These data suggest that the feedback projections from the amygdala to the auditory cortex have a frequency-related, topographic organization.

## Discussion

The amygdala complex is important for associating affective information with sensory information. On the anatomical ground that La receives afferents from a wide range of locations in the thalamus and sensory cortex, auditory cues and footshock information converge in La^21^ and are associated with one another based on synaptic plasticity^34,35^. Therefore, La is considered to be the center to the establishment of fear conditioning^11^. The associated information in La is then sent to CeA, which is indispensable for expressing freezing behaviors^36^. The output from CeA is then sent to a wide range of brain regions including the bed nucleus, hippocampus, midbrain, pons, and medulla oblongata^11,18^. This general scheme illustrates La as the input center and CeA as the output center of the amygdala. However, sensory cortices, including the auditory cortex, have not been found to be output targets of CeA. Indeed, old tracing studies had already revealed that the auditory cortex receives dense projections from the lateral parts of the amygdala in rats^28^ and primates^27^. Both our findings and those of a recent study^24^ confirm the presence of direct projections from La to the auditory cortex in mice, as well as the absence of projections from CeA to the auditory cortex. A similar scheme is also applicable to the visual system. The postrhinal area (POR) is a higher order visual cortical area that receives direct feedback projections from La but not from CeA in mice, and is modulated by La during processing of motivationally relevant sensory cues^37^. Therefore, it is reasonable to classify La, and not the CeA, as the main center in the amygdala that outputs information to the sensory cortex.

The results of the current study indicate that A2 specifically outputs and inputs signals to/from La. Although previous studies have reported evidence of a direct feedback pathway from La to the auditory cortex both in mice^24,25^ and rats^28^, whether the entire auditory cortex is uniformly innervated by La has not been determined. As for the feedforward pathway from the auditory cortex to the amygdala, a consensus has not been made regarding whether the projections originate throughout the entire auditory cortex or are localized. Interestingly, one tracing study suggested that the ventral parts in the rat auditory cortex give rise to projections towards the amygdala^14^. However, the stereotaxic identification of the auditory cortex in this study was based on the classical regional annotation of Te1, Te2, and Te3^38^, which is quite different from the modern classification using AAF, A1, and A2. In the current study, the combination of neuronal tracing with prior functional identification of the auditory subregions using optical imaging enabled us to reveal the precise projectional topography based on the modern map of the auditory cortex. Thus, this study is the first to show that both the feedforward and feedback pathways between the auditory cortex and amygdala do not uniformly originate or terminate across the entire auditory cortex or amygdala in mice, and that A2 and La are main conduits for communication between the auditory cortex and amygdala.

In the current study, injections were conducted according to the auditory cortex map that had been established by our previous reports^4,6,30,31,39–41^. However, the exact definition of the mouse auditory cortical regions is still under debate^29,42^, and interpretation of the current results could be differentiated depending on how the auditory cortex is mapped. Disagreements regarding the macroscale auditory cortical structure are derived from two major sources. First, it has not been determined whether the tonotopic direction of AAF is dorsoventral or ventrodorsal. The original map draws AAF as it runs ventrodorsally^2^, which influenced several following studies using electrophysiology^1^ and voltage-sensitive-dye imaging^43^. In contrast, other studies, including our observations, draw AAF as it runs dorsoventrally using flavoprotein fluorescence imaging^4,44^, GCaMP3^3^, GCaMP6 imaging^42^, and intrinsic signal imaging^45^. A recent study attempted to reconcile these opposite results by proposing that AAF has branched tonotopy with two high frequency targets^29^. Although the precise location of the high frequency part of AAF remains unclear, all the studies identify the same location as the low frequency part of AAF. In the current study, we injected CTB into the low frequency part of AAF; therefore, the current results regarding injection into AAF can be generalized across different auditory cortex maps. Second, it has not been determined whether the tonotopic regions of A1 and DM are different regions or a single region. The original study^2^, electrophysiology^1^, and voltage-sensitive-dye imaging studies^43^ identify one tonotopic region of A1 in the caudal auditory cortex. Studies using GCaMP3^3^ and GCaMP6 imaging^42^ suggest the possibility that the A1 tonotopy is branched as it runs from one low frequency part towards two high frequency parts. Our previous studies suggest that two branches in A1 can be split into two distinct subregions and named the dorsal region DM and the ventral region A1^46^. Intrinsic signal imaging studies agree with our findings although name these two subregions A1 and the ventral auditory field (VAF), respectively^47^. If there are not two separate regions, but rather one single causal region, the current results would be unchanged. CTB-positive neurons for A1 and DM would be combined, and the mean number of CTB-positive neurons found in the amygdala per animal is changed to 319.6 (A2 injection), 42.3 (“enlarged A1” injection), and 3.0 neurons (AAF injection), from 319.6 (A2 injection), 81.0 (A1 injection), 3.0 (AAF injection), and 3.5 neurons (DM injection). Even after conducting this reanalysis, the number of CTB-positive axons and somata in the amygdala are significantly larger when injecting CTB into A2. Therefore, predominance of A2 over other auditory subregions in terms of connectivity with the amygdala is not changed depending on definition of the auditory cortex map. The current study was conducted based on the auditory cortex map that had been described in our previous studies, where A1 was assigned to the second most ventral part. However, it is more straightforward that the region annotated as A1 sits in the location with the shortest latency. The recent find-grained mapping using optical imaging has paved the way for the possibility that the auditory cortex map can be comparable between mice and rats; therefore, despite debate over defining auditory subregions, the terminology could be unified between mice and rats in future.

Overall, our findings endorse the notion that A2 is a higher-order auditory region in the mouse auditory cortex. The auditory subregions of DM, AAF, A1, and A2 have been found to be tonotopic^4^, and all of these fields receive substantial projections from the lemniscal auditory thalamus of the ventral division of MGB (MGv)^4,40^. Because the general concept explains that cortical areas that receive afferents from MGv are primary auditory areas, a question arises regarding whether these fields, including A2, are all primary areas in the mouse cortex. However, the results of the current study clearly show the presence of dense connections between A2 and the amygdala, while other auditory fields have slight or no connections with the amygdala. Moreover, previous studies have reported that A2 neurons have long latencies^1,30,48^, wide bandwidth^3,40^, and a less-ordered tonotopic arrangement^3,40^. These data confirm that mouse A2 has properties of a higher-order region, and also support the idea that auditory cortical subregions are functionally specialized in mice^5,6,49^. Given that primary-like regions have few feedforward or feedback connections with the amygdala, they may work to analyze auditory information, as previously proposed. Although our data showed a relatively long latency in the region labeled A1, the connectivity with the amygdala was faint, indicating that A1 may function as a primary-like region. In contrast, A2 may work in cooperation with the amygdala to associate affective information with auditory information after it receives auditory information from primary fields via the corticocortical pathways^50,51^. A recent study using rats suggested that value-coding neurons exist in parts that correspond to those located near mouse A2^52,53^, which may be evidence of the La–A2 feedback pathways. It is possible that A2 works to provide biological meaning for emotion-arousing sounds such as conspecific courtship songs and predatory calls, and thus plays a more important role in signal processing than simple sound analysis.

## Methods

### Animals

The experimental procedures in the present study were approved by the Committee for Animal Care at Niigata University (Protocol Number: SA00235), and carried out in accordance with the approved guidelines. Data were obtained from male C57BL/6 mice at the age of 7–8 weeks (Charles River Japan, Kanagawa, Japan). The animals were housed in cages with *ad libitum* access to food pellets and water, and were kept on a 12-h light/dark cycle.

### Flavoprotein fluorescence imaging

Locations of regions in right auditory cortex were identified according to responses revealed using flavoprotein fluorescence imaging^54^. Cortical images were recorded by a CCD camera system (AQUACOSMOS with ORCA-R2, Hamamatsu Photonics, Shizuoka, Japan) via an epifluorescence microscope (Ex, 500–550 nm; Em, 470–490 nm; M651 combined with MZ FL II, Leica Microsystems, Wetzlar, Germany). The area covered by one pixel was 20.4 × 20.4 µm. Mice were anesthetized with urethane (1.65 g/kg, i.p.; Wako, Osaka, Japan). The rectal temperature was maintained at ~37 °C. A craniotomy (~3 × 3 mm) was performed over the right auditory cortex. The auditory cortex was activated by presentation of sound waves that were made using a LabVIEW program (National Instruments, Austin, TX). Tones were amplitude modulated (20 Hz, 100% modulation) and set to ~60 dBSPL. The location of auditory cortical subregions was identified according to the tonotopic shift that were visualized using 5 and 30 kHz tones.

### Neural tracer injection

A glass pipette (tip diameter ~30 μm) filled with cholera toxin subunit b (CTB) solution was introduced into the center of auditory regions visualized and identified using optical imaging, to ~450 μm below the cortical surface so that tracer solutions spread to all the cortical layers. Here, we used a low-salt type CTB (#104; List Biological Laboratory, Campbell, CA) that is suitable for iontophoretic injection^55^. CTB was injected by carrying 70 pulses, 5 s on/5 s off anodal currents at an intensity of 4 μA. Partly, Alexa Fluor 488- and 555-conjugated CTBs (0.5% in 0.1 M phosphate buffer, Thermo Fisher Scientific, Waltham, MA) were iontophoretically injected in A1 and A2 each in the same animals by applying 70 pulses. After finishing injections, a glass pipette was slowly withdrawn. The cranial hole was covered using 2% agarose (1-B, Sigma-Aldrich, MO) and the skin was sutured. Mice were placed in a warm place for recovery, and after awaking they were reared in their home cages. In several experiments, we injected a 10% biotinylated dextran amine (BDA, mw: 3,000, Thermo Fisher Scientific) solution by carrying ~40 pulses, 7 s on/7 s off anodal currents at an intensity of 5 μA via a ~20 μm-thick pipette.

### Histology

Three days after CTB injections, mice were anesthetized with an overdose of pentobarbital (0.3 g/kg, i.p.), and cardiac perfusion perfused transcardially with 4% paraformaldehyde. Brains were removed and immersed in 4% paraformaldehyde overnight. After incubated in 20% and 30% sucrose in 20 mM phosphate buffered saline (PBS), consecutive 40 µm-thick coronal sections were made using a sliding cryotome (REM-710, Yamato-Koki, Saitama, Japan). Every fourth slice was used for analysis.

To visualize CTB, sections were initially rinsed in 20 mM PBS and incubated in PBS containing 3% hydrogen peroxide and 0.1% Triton X-100 for 15 min at room temperature. After incubating in 20 mM PBS containing 0.1% Triton X-100 (PBST) for 60 min, slices were incubated overnight at room temperature with goat anti-CTB antibody (List Biological Laboratories) diluted to 1:30,000 with 20 mM PBS containing 0.5% skim milk. The next day, slices were rinsed in 20 mM PBS, and incubated at room temperature for 2 h with HRP-conjugated rabbit anti-goat IgG antibody (MBL, Nagoya, Japan) diluted to 1:200 using 20 mM PBS containing 0.5% skim milk. Sections were rinsed in 20 mM PBS, and the immunoreactions were visualized in a Tris-HCl buffer containing 0.05% diaminobenzidine tetrahydrochloride and 0.003% hydrogen peroxide for 5 min at room temperature. After visualization, slices were Nissl-stained using 0.1% cresyl violet (Chroma Gesellschaft, Kongen, Germany), and they were dehydrated in ethanol, cleared in xylene, and cover-slipped using the covering reagent Bioleit (Okenshoji, Tokyo, Japan). When coverslipping sections with fluorescent CTB, Fluoromount (Cosmo Bio, Tokyo, Japan) was used as a covering reagent instead. To visualize BDA, slices were incubated in a Tris-HCl buffer containing 0.05% diaminobenzidine tetrahydrochloride and 0.003% hydrogen peroxide for 20 min.

Borders of subdivisions in the amygdala were drawn according to the mouse brain atlas^56^ and Nissl staining patterns. Histological images were obtained using a CCD camera (DP80; Olympus, Tokyo, Japan) via a stereoscopic microscope (Eclipse Ni, Nikon, Tokyo, Japan) using white light or emission filters (515–555 nm for greed and 600–660 nm for red).

### Statistics

The Mann-Whitney U test or Wilcoxon signed-rank test was used to evaluate differences between unpaired or paired data from the two groups, respectively. If multiple comparisons were in need, p-values were corrected using the Bonferroni correction. The Dunnett’s test was used to evaluate multiple data against reference data. The chi-squared test was used to evaluate differences between two distributions. All tests were conducted as a two-sided test using MATLAB programs (Mathworks, St. Louise, MO) or SPSS (IBM, Armonk, NY). All data in graphs are presented as mean ± S.E.M.

## Supplementary information

Supplemental Information

## Data Availability

The datasets are available from the corresponding authors on reasonable request.
