# Supplementary material for: Reciprocal connectivity between secondary auditory cortical field and amygdala in mice
Source: Sci Rep. 2019 Dec 23;9:19610. doi: 10.1038/s41598-019-56092-9 (PMC6928164; doi:10.1038/s41598-019-56092-9)
Supplement: Supplementary file 1 — Supplemental Information [file 41598_2019_56092_MOESM1_ESM.docx]

**Supplemental Information**

**Reciprocal connectivity between secondary auditory cortical field and amygdala in mice**

Hiroaki Tsukano^1,2*^, Xubin Hou^3^, Masao Horie^4^, Hiroki Kitaura^5^, Nana Nishio^2,6^, Ryuichi Hishida^2^, Kuniyuki Takahashi^7^, Akiyoshi Kakita^5^, Hirohide Takebayashi^8^, Sayaka Sugiyama^3^, Katsuei Shibuki^2^

1. Department of Psychiatry, The University of North Carolina at Chapel Hill, 116 Manning Drive, Chapel Hill, NC, 27599, USA
2. Department of Neurophysiology, Brain Research Institute, Niigata University, 1-757 Asahimachi-dori, Chuo-ku, Niigata 951-8585, Japan
3. Laboratory of Neuronal Development, Graduate School of Medical and Dental Sciences, Niigata University, 1-757 Asahimachi-dori, Chuo-ku, Niigata 951-8510, Japan
4. Department of Nursing, Niigata College of Nursing, 240 Shinnancho, Joetsu, 943-0147, Japan
5. Department of Pathology, Brain Research Institute, Niigata University, 1-757 Asahimachi-dori, Chuo-ku, Niigata 951-8585, Japan
6. Department of Physiology, The University of Tokyo School of Medicine, 7-3-1 Hongo, Bunkyo-ku, Tokyo 113-0033, Japan
7. Division of Otolaryngology, Graduate School of Medicine and Dental Sciences, Niigata University, 1-757 Asahimachi-dori, Chuo-ku, Niigata 951-8510, Japan
8. Division of Neurobiology and Anatomy, Graduate School of Medicine and Dental Sciences, Niigata University, 1-757 Asahimachi-dori, Chuo-ku, Niigata 951-8510, Japan

**Supplemental Figures**


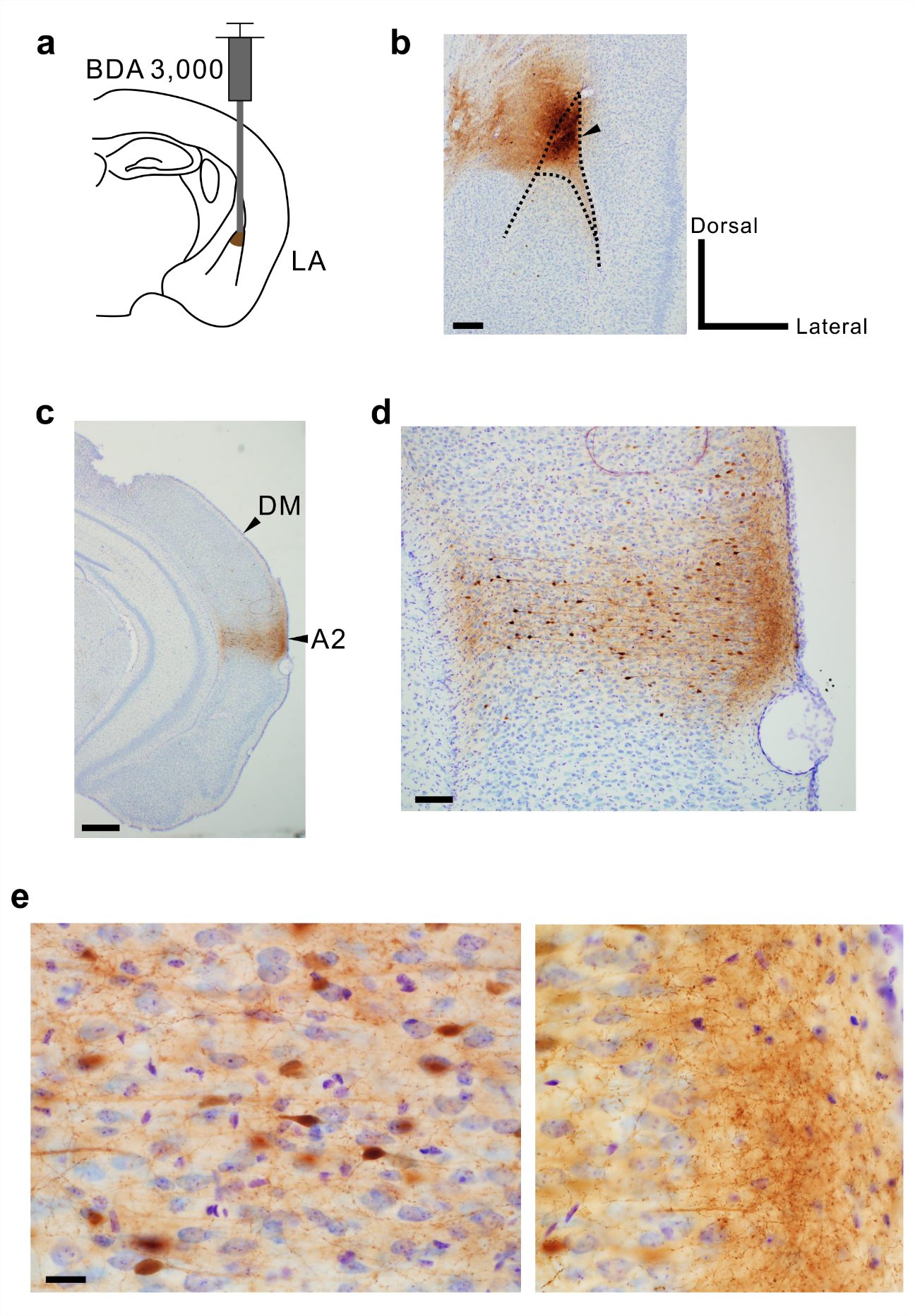


**Supplemental Figure 1.** An example image of auditory cortex after injecting BDA into LA. (**a**) Illustration showing injection of BDA into LA. (**b**) LA after injection of BDA. In the separate experiments where BDA is injected into the Striatum, there exist no axon terminals inside of the auditory cortex (data not shown) as generally thought. Scale bar, 200 μm. (**c**) A low magnification image of A2 after injecting BDA into LA. The arrow heads indicate the location of A2 and DM that are identified according to the previous study^1^. Scale bar, 500 μm. (**d**) A magnified image of A2 in (**c**). Scale bar, 100 μm. (**e**) High magnification images of A2. Axon terminal buttons in layer 2/3 (left) and layer 1 (right) that project from LA are shown. Scale bar, 20 μm.


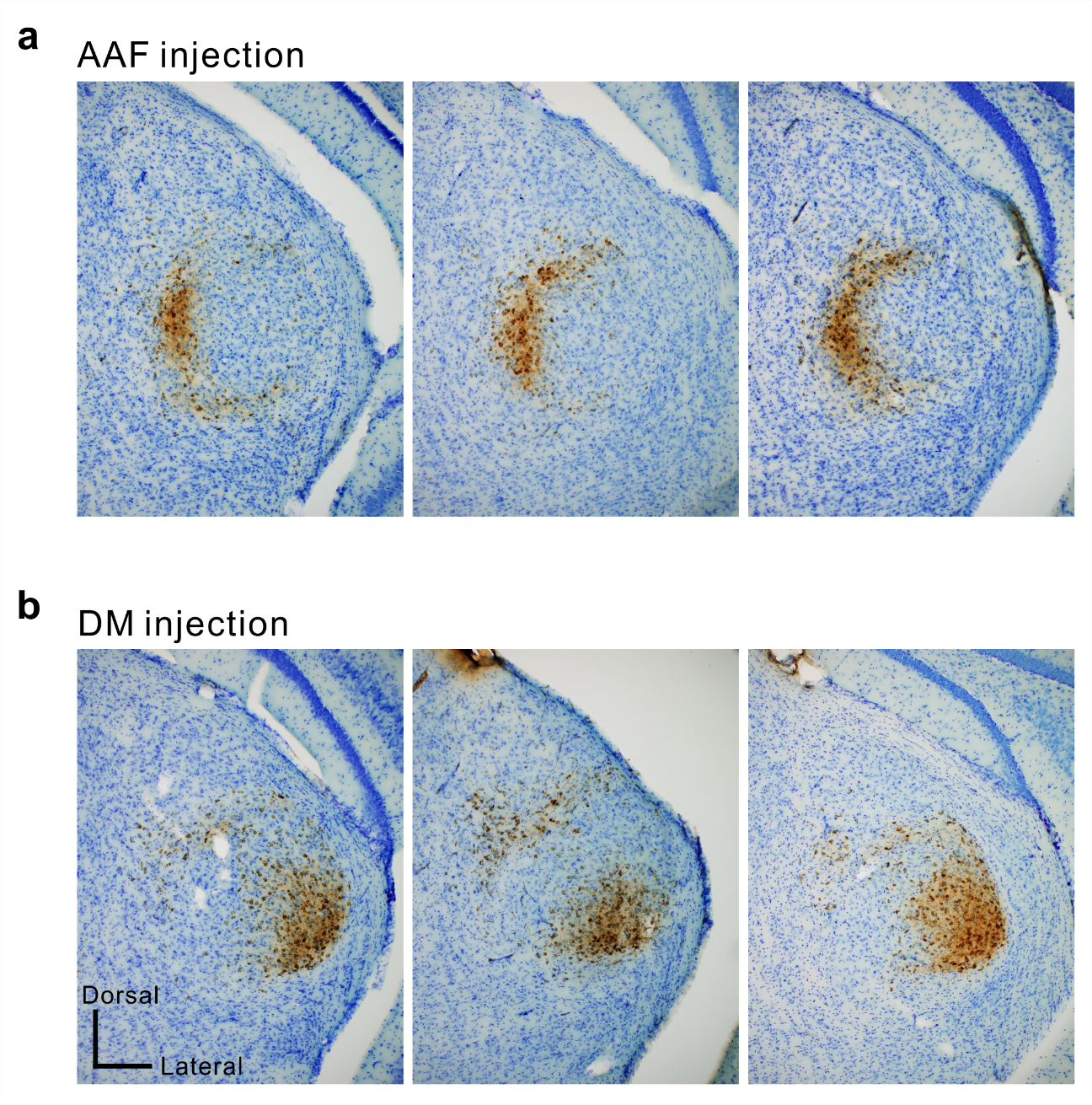


**Supplemental Figure 2.** CTB-positive neurons in the auditory thalamus. (**a**) Neurons that are retrogradely stained with CTB after injecting CTB into AAF. Three images show the auditory thalamus from different three mice. (**b**) Neurons that are retrogradely stained with CTB after injecting CTB into DM. Three images show the auditory thalamus from different three mice. These results are consistent with the previous studies^2,3^. Scale bar, 200 μm.

**References**

1. Tsukano, H. et al. Quantitative map of multiple auditory cortical regions with a stereotaxic fine-scale atlas of the mouse brain. *Sci. Rep.* **6**, 22315 (2016).
2. Horie, M., Tsukano, H., Hishida, R., Takebayashi, H. & Shibuki, K. Dual compartments of the ventral division of the medial geniculate body projecting to the core region of the auditory cortex in C57BL/6 mice. *Neurosci. Res.* **76**, 207–212 (2013).
3. Tsukano, H. et al. Independent tonotopy and thalamocortical projection patterns in two adjacent parts of the classical primary auditory cortex in mice. *Neurosci. Lett.* **637**, 26–30 (2017).
